# The high-quality telomere-to-telomere genome assembly of the earthworm (*Amynthas aspergillum*)

**DOI:** 10.1038/s41597-025-05058-w

**Published:** 2025-06-02

**Authors:** Guangquan Peng, Yanghe Qin, Zhiming Yan, Mingwei He, Yucheng Zhao, Neng Jiang, Changhong Wei

**Affiliations:** 1 Department of Research & Clinical Laboratory, The Fifth Affiliated Hospital of Guangxi Medical University & The First People’s Hospital of Nanning, Nanning, China; 2 Department of Pharmacy, Guangxi Medical University Cancer Hospital, Nanning, 530021 Guangxi PR China; 3 State Key Laboratory of Natural Medicines, Department of Resources Science of Traditional Chinese Medicines, School of Traditional Chinese Pharmacy, China Pharmaceutical University, Nanjing, 211198 China; 4 Institute for Safflower Industry Research, Key Laboratory of Xinjiang Phytomedicine Resource and Utilization (Ministry of Education), School of Pharmacy, Shihezi University, Shihezi, 832002 China

**Keywords:** Genome assembly algorithms, Bioinformatics

## Abstract

Earthworms have been extensively studied as ancient soil invertebrates, that are highly diverse. Previous studies of these invertebrates have mainly focused on their ecosystem services, medicinal value, and ecological habits. However, their genomic analysis remains inadequate. In this study, we generated the first high-quality telomere-to-telomere (T2T) assembly of the genome of the earthworm, *Amynthas aspergillum (Perrier, 1872)*, which belongs to the genus *Amynthas* of the family Megascolecidae. The T2T assembly was 758.86 Mb with an N50 contig size of 16.59 Mb. The sequences were anchored to 43 chromosomes (2n = 2x = 86) with a coverage of 98.43% (746.95 Mb), and 83 telomeres were detected. In addition, we also predicted 35,723 protein-coding genes with 97.02% being functionally annotated. This T2T genome assembly will establish substantial groundwork for exploring the evolutionary mechanisms of the earthworm genome and enhance the specificity of its pharmacological effects.

## Background & Summary

Earthworms, classified under the Oligochaeta genus within the phylum Annelida, are ancient terrestrial invertebrates with significant ecological and medicinal importance^1^. There are more than 3,000 species of earthworms worldwide, with more than 600 species found in China alone^2^. These organisms play a pivotal role in soil ecosystems by decomposing organic matter, thereby enhancing microbial activity and soil fertility^2,3^. Their ecological contributions extend to waste management and environmental remediation, with applications in sewage purification and soil quality improvement^3–5^. Moreover, earthworms are used in traditional Chinese medicine to treat various diseases such as high fever, dizziness, joint paralysis, and urinary edema^6^, and they are also used as an emerging high-protein feed.

**Fig. 1:**
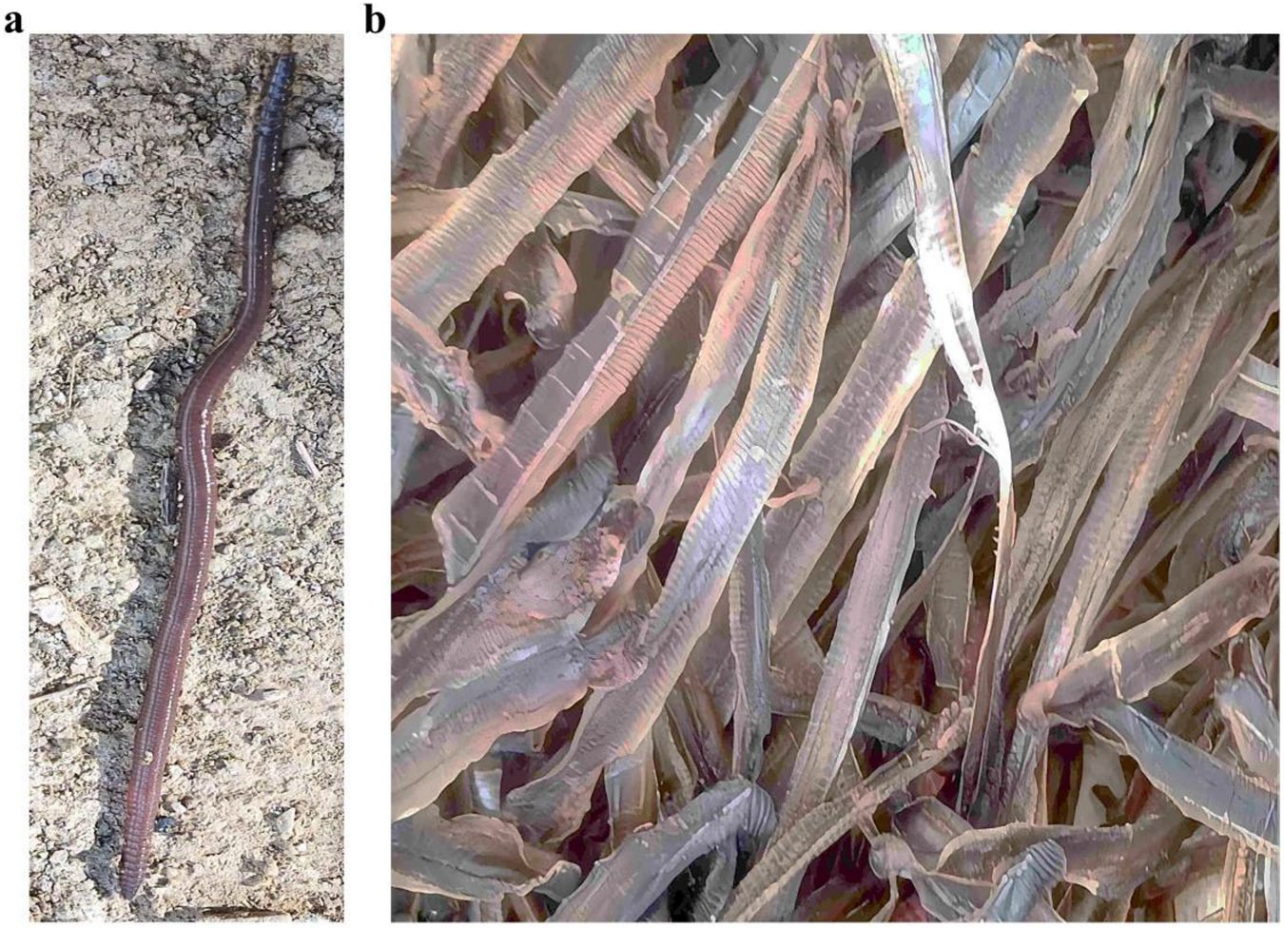
Photograph of *A. aspergillum*. (**a**) A live sub-adult earthworm. (**b**) The dried bodies used for medicinal purposes.

*Amynthas aspergillum (Perrier, 1872)*, also known as geosaurus or “Guang dilong”, is a terrestrial annelid belonging to the genus *Amynthas* in the family Megascolecidae. It is usually 15–20 cm long and 1-2 cm wide (Fig. 1a). This species is predominantly found in Chinese provinces such as Guangxi, Guangdong, and Fujian^6^. As a traditional Chinese medicine, the earthworm first appeared as the “white-necked earthworm” in *Shennong Bencao Jing* of the Eastern Han Dynasty, and it began to be called “Guang dilong” in the *Revised Materia Medica* of the Tang Dynasty^7^. *A. aspergillum* is one of 10 varieties of geo-authentic traditional Chinese medicine in Guangxi and has been widely used in traditional Chinese medicine for thousands of years because of its excellent medicinal properties. Its medicinal form is the dried body (Fig. 1b), and its efficacy is better than that of “Hu dilong” and “Tu dilong”^7^. In addition, *A. aspergillum* is widely used in modern clinical medicine, and studies of its chemical composition and pharmacological activity were conducted as early as 1974^8^. It contains diverse chemical components, of which polypeptides, nucleosides, lipids, enzymes, and amino acids are the main active ingredients^9^. These components underpin its diverse pharmacological properties, including anti-tumor, anti-thrombotic, anti-hypertensive, immunomodulatory, and wound-healing effects^10–12^.

In recent years, with increasing research on earthworms, progress has been made on species identification, chemical composition, pharmacological effects, and quality control^5,13^, but few studies have investigated earthworm genomics. The lack of high-quality genomes and further research on earthworm genomics seriously hinders our in-depth understanding and exploration of their functions and evolutionary processes. To date, the chromosome-level genomes of six earthworm species (*Eisenia fetida*^14^, *Eisenia andrei*^15^, *Metaphire vulgaris*^16^, *Amynthas corticis*^17^, *Lumbricus rubellus*^18^, and *Lumbricus terrestris*^19^) have been sequenced and assembled, laying a foundation for studies of earthworm ecology, evolutionary mechanisms, and molecular mechanisms of immune defense and earthworm regeneration^20^. However, this is insignificant compared to the large number of earthworms species. Notably, no telomere-to-telomere (T2T) genome has been assembled to date, leaving significant gaps in our knowledge of their genomic architecture and evolutionary mechanisms. The availability of genomes from more species will provide valuable insights into the genetics and molecular mechanisms of earthworms.

Here, we sequenced and assembled the first complete T2T genome of the earthworm *A. aspergillum* by combining Pacific Biosciences (PacBio) HiFi, Oxford Nanopore Technologies (ONT) ultra-long, Illumina, and high-throughput chromosome conformation capture (Hi-C) sequencing technologies. Sequencing data and completeness assessments suggested that the T2T assembly was superior to the previous chromosome-level assembly. The assembly demonstrates exceptional quality, with an N50 contig length significantly exceeding those of previously sequenced earthworm genomes. In summary, we utilized multiple methods combining genetics and cytology to produce a high-quality T2T genome that will bring new opportunities to identify the unique genes and structural variations in the “dark matter” regions, such as centromeres, transposable elements and segmental duplications.

## Methods

### Sample collection and pre-treatment

One sexually mature *A. aspergillum* was gathered from a breeding base that is located in Longan Town, Nanning, Guangxi, China (107°47′59′′E, 23°4′29′′). The sample has been sequenced by COI gene sequencing and identified as *A. aspergillum* with 100% species similarity (Fig. S1). After rinsing with saline solution to remove any attached dirt, the earthworm was dissected to remove its gut. Then the body wall was washed thoroughly three times using 1 × PBS and was carefully sheared into tissues less than 0.5 cm both in length and width. Eventually, the pre-treatment sample was stored at −80 °C and would be used for further DNA extraction and sequencing.

### DNA extraction and sequencing

The body tissue of *A. aspergillum* was used for high-quality DNA isolation, and then the PacBio HiFi library, ONT library, Illumina library, and Hi-C library were constructed following the manufacturer’s instructions. Briefly, the genomic DNA was damaged, end-repaired, ligated to adapters, and exonuclease digested. Then the digested DNA was screened for target fragments using BluePippin to obtain the PacBio HiFi library, which was sequenced on the PacBio Revio platform and switched to CCS (Circular Consensus Sequence) data using the smrtlink v9.0 of PacBio program. For ultra-long ONT sequencing, a library was generated with the Oxford Nanopore SQK-ULK001 kit following the standardized protocol and then sequenced on the PromethION platform. The Illumina library was constructed through DNA breaking, end repairing, adding A tail, ligating adapters, selecting target fragments, and expanding with PCR. After using Qseq. 400 and Qubit to detect fragment size and quality, the library was sequenced on the Illumina NOvaSeq. 6000 platform (PE150). As a result, we generated 71.00 Gb (~92×) Illumina short reads, 80.34 Gb (~101×) of CCS reads with an average length of 16.21 kb, 25.75 Gb (~30×) of ultra-long ONT reads with an average length of 97.68 kb (Table S1).

For Hi-C sequencing, the experiment type of library construction was *in situ* Hi-C, including crosslinking DNA, cutting with restriction enzyme Hind III, filling ends and marking with biotin, ligating, purifying, and shearing DNA into 300 bp~700 bp fragments and pulling down biotin. The concentration and insert size of the library were detected by Qubit2.0 and Agilent 2100, respectively. Next, the library was sequenced by using the Illumina NOvaSeq. 6000 platform (PE150)^21^. Finally, 130.81 Gb (~171×) of Hi-C reads were generated (Table S1). All DNA isolation, library construction, and gene sequencing procedures were processed by the BIOMARKER Company (Beijing, China) according to the manufacturer’s protocols.

### Genome survey, *de novo* genome assembly, and telomeres identification

**Fig. 2:**
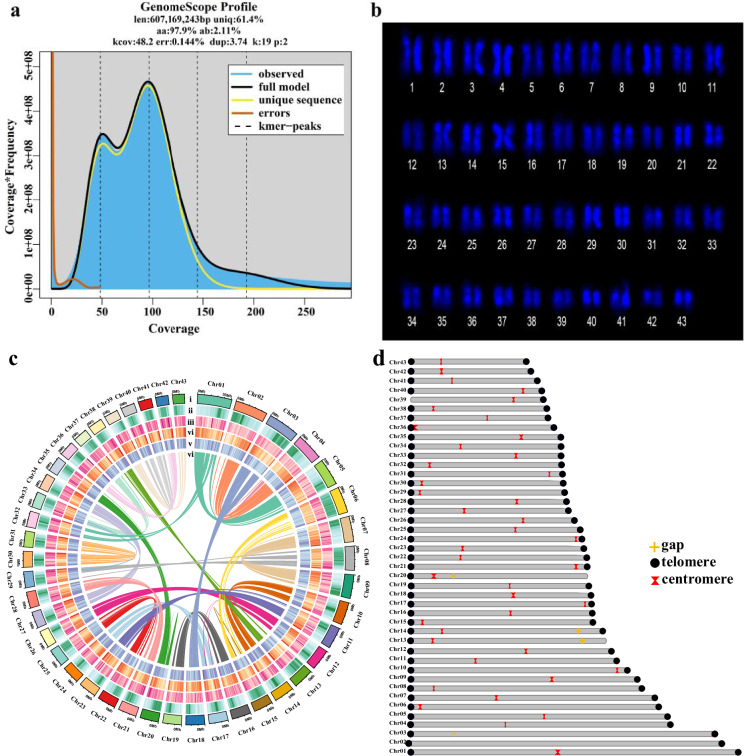
Genome and chromosome features of *A. aspergillum*. (**a**) K-mer distribution (k = 19) in the *A. aspergillum* genome. The graph serves as an indicator of the estimation of genome complexity and provides a strategy for subsequent genome assembly. (**b**) The image of each chromosome in one individual *A. aspergillum* produced in karyotype analysis (2n = 2x = 86). (**c**) Genome characteristics of *A. aspergillum*. Circos plot from the outer to the inner layers represents the following: (i) Pseudo-chromosomes (Chr1-Chr43); (ii) TEs density; (iii) SSR density; (iv) Gene density; (v) GC content; (vi) Syntenic blocks within the *A. aspergillum* genome. (**d**) The distributions of chromosome features. The numbers on the left are the numbers of each chromosome (Chr1-Chr43). The circle in yellow and the triangle in black represent gaps and telomeres of chromosomes in the genome, respectively. The crossover site in each chromosome represents centromeres.

A genome survey of *A. aspergillum* based on 19 K-mer frequencies of Illumina short reads using jellyfish^22^ v2.1.4 (-h 1000000000) and Genomescope^23^ v2.0 (-k 19 -p 2 -m 100000) indicated that the genome was approximately 607.17 Mb with a high level of repetitive sequence content (~38.64%) and heterozygosity (~2.11%) (Fig. 2a). The results showed that the genome of *A. aspergillum* is highly heterogeneous and complex, and we speculated that the earthworm is diploid, which is consistent with the results of the karyotype analysis (2n = 2x = 86) (Fig. 2b and S2).

All of the sequencing data were subjected to quality control to filter out adapter sequences and low-quality reads, ensuring that clean data were generated. With the CCS, ultra-long ONT, and Hi-C high-accuracy data, initial contig assembly was generated using hifiasm v0.19.5-r587 (hifiasm -t40–ul). After removing plasmids and contaminating sequences, the contigs were clustered, ordered, and oriented onto chromosomes based on the high-quality Hi-C data by using LACHESIS^24^ v2.0.1 (CLUSTER_MIN_RE_SITES = 342; CLUSTER_MAX_LINK_ DENSITY = 2; ORDER_MIN_N_RES_IN_TRUNK = 652; ORDER_MIN_ N_RES_IN_SHREDS = 634), generating a chromosome-level genome. The filtered CCS and ONT reads were initially assembled into 205 contigs with a total length of 758.86 Mb, an N50 of 16.59 Mb, and the largest length of 30.87 Mb (Table 1). A total of 746.95 Mb (98.43%) of genomic sequences were anchored to 43 chromosomes (Fig. 2c,d), and the largest chromosome reached a length of 30.87 Mb (Table S2). Among the sequences anchored to the chromosomes, the length of the sequences that could determine the order and orientation was 746.95 Mb, accounting for 100.0% of the anchored sequences. Furthermore, we identified 72 telomeres, 43 centromeres, and 5 gaps in the assembled chromosome-level genome using TIDK (https://github.com/tolkit/ telomeric-identifier), FindTelomeres (https://github.com/JanaSperschneider/ FindTelomeres), and Centromics software (https://github.com/ShuaiNIEgithub/ Centromics), of which 14 lacked telomeres at single ends and only 38 chromosomes were gapless T2T chromosomes.

**Table 1:** Basic information on genome assembly in earthworms.

Assembly	*A. aspergillum*	*E. fetida*	*E. andrei*	*M. vulgaris*	*A. corticis*	*L. rubellus*	*L. terrestris*
Total length	758.86 Mb	1.47 Gb	1.3 Gb	728.57 Mb	1.19 Gb	787.5 Mb	1.06 Gb
Contig number	205	463,133	2,970	559	16,882	2,261	1,587
Contig N50	16.59 Mb	967 kb	740 kb	4.20 Mb	117 kb	0.7 Mb	1.6 Mb
GC content	40.76%	40%	35–45%	40%	40.34%	40.5%	40.8%
Complete BUSCOs	95.18%	18.2%	92.1%	94.3%	91.2%	90.6%	91.4%
Protein-coding genes	35,723	—	31,817	—	29,256	33,426	—

Subsequently, the ultra-long ONT data was used for filling gaps via TGS-GapCloser^25^ and quarTeT software, and chromosomes missing telomeric regions were aligned to the ends of chromosomes using ultra-long ONT reads to extend the telomeric regions. The transcriptomic data also assisted in optimizing genome structure in three ways: identification of exon-intron boundaries, discovery of new exons and variable splicing, and discovery of non-coding RNAs and UTR regions. Eventually, we obtained a complete T2T genome of *A. aspergillum*, where a total of 83 telomeres, 43 centromeres, and only 4 gaps were identified. It was worth noting that telomeres were detected on both ends of 40 chromosomes, and 38 chromosomes (88.4%) were gapless T2T chromosomes (Fig. 2d and Table S2). Compared to the previous chromosome-level assembly of earthworm, *A. aspergillum*, the first T2T earthworm genome showed significant improvements in genome accuracy and continuity (Table 1).

### Repeat element annotation

Transposon elements (TEs) and tandem repeats were annotated by the following methods. TEs were identified by combining homology-based and *de novo* approaches^26^. We first customized a *de novo* repeat library of the genome using RepeatModeler^27^ v2.0.1 (BuildDatabase -name & & RepeatModeler -pa 12), which can automatically execute two *de novo* repeat finding programs, including RECON^28^ v1.0.8 and RepeatScout^29^ v1.0.6. The long terminal repeat retrotransposons (LTR-RTs) were identified using both LTRharvest^30^ v1.5.10 and LTR_FINDER^31^ v1.07. The high-quality intact LTR-RTs and non-redundant LTR library were produced by LTR_retriever^32^ V2.9.0 with default parameters and used to examine the insertion time of LTR-RTs. Subsequently, a non-redundant species-specific TE library was developed by integrating the *de novo* TE sequences library above with the known Dfam v3.5 database. The final TE sequences in the *A. aspergillum* genome were identified and classified through a homology search against this library using RepeatMasker v4.1.0^33^ (repeat masker -nolow -no_is -norna -engine wublast -parallel 8 -qq). Moreover, we also identified tandem repeats, including microsatellites, minisatellites, and satellites, using the MIcroSAtellite identification tool (MISA)^34^ v2.1 and Tandem Repeat Finder (TRF)^35^ v409 (2 7 7 80 10 50 500 -d -h). As a result, approximately 49.33% (374,336,823 bp) of the *A. aspergillum* genome sequences were identified as repetitive, of which 39.49% were transposable elements (TEs) and 9.84% were tandem repeats. Among these, long interspersed nuclear elements (LINEs), short interspersed nuclear elements (SINEs), and long terminal repeat retrotransposons (LTR-RTs) accounted for 7.76%, 0.32%, and 11.44% of the genome, respectively (Table 2).

**Table 2:** Statistics of repeat annotations of *A. aspergillum*.

Type		Sequence length (bp)	Percentage of the genome (%)
Transposable elements	LINEs	58,870,972	7.76
	SINEs	2,441,270	0.32
	LTR-RTs	86,816,877	11.44
	DNA transposon	151,509,405	19.97
	Other	2,088	0
	Unknown	32,312	0
Tandem repeats	microsatellite (1–9 bp units)	28,471,601	3.75
	minisatellite (10–99 bp units)	20,117,127	2.65
	satellite (> = 100 bp units)	26,075,171	3.44
Total		374,336,823	49.33

### Gene prediction and genome functional annotation

In addition to the repeat sequences, we predicted 35,723 protein-coding genes from the repeat-masked genome of *A. aspergillum* through a combined strategy of *de novo*, homologous, and RNA-sequencing-based predictions (Table 1 and S3). Specifically, *de novo* prediction was performed by using Augustus^36^ v3.1.0 and SNAP^37^ (2006-07-28) with default parameters. For homology-based prediction, we utilized Miniport v1.7 (run. sh mmseqs) to determine a comparative analysis of the sequences from model organisms and closely related species, including *Caenorhabditis elegans*^38^, *A. corticis*, *E. andrei*, and *L. terrestris*. These sequences were downloaded from the National Center for Biotechnology Information (NCBI) database and compared to the *A. aspergillum* genome to predict gene structure according to homology-based evidence. Moreover, we extracted total RNA and generated RNA reads with a total of 10.21 Gb of clean data from the body tissue of *A. aspergillum* (Table S4). Then GeneMarkS-T^39^ v5.1 and PASA^40^ v2.4.1 with default parameters were used for transcriptome-based prediction with the RNA-seq clean data. Finally, the prediction results obtained from the above three methods were incorporated using EVidenceModeler (EVM)^41^ v1.1.1 with default parameters and modified using PASA^40^ v2.4.1 to generate the final coding gene set. In contrast, 3,697 noncoding RNAs, 138 pseudogenes whose biological functions were lost, 1,959 conserved motifs, and 65,549 domains were identified based on the respective annotation method (Table S5).

After gene prediction, we conducted gene functional annotation by aligning the protein-coding gene sequences obtained from the preceding methods against the Non-Redundant (NR)^42^, EggNOG^43^, TrEMBL^44^, KOG, SWISS-PROT^44^ and Pfam^45^ protein databases using diamond v0.9.29.130 (diamond blastp–masking 0 -e 0.001) and the Kyoto Encyclopedia of Genes and Genomes (KEGG)^46^ database (http://www.genome.jp/kegg/) with an E-value threshold of 1E-3. Gene Ontology (GO)^47^ IDs (http://www.geneontology.org/) for each gene were obtained from TrEMBL^44^, InterPro^48^, and EggNOG^43^. A total of 31,657 protein-coding genes were annotated, accounting for 88.62% of all predicted genes in *A. aspergillum*. The specific functional annotation statistics are presented in Table S3. In the eggNOG function classification, the unknown function group (S) accounted for the largest proportion, reaching approximately 23.88% (Fig. S3).

## Data Records

All sequencing raw data have been deposited into the NCBI Sequence Read Archive (SRA) database at SRR31656544-SRR31656548^49–53^. In addition, the T2T Genome data have been deposited at the NCBI database under the accession JBJUSN000000000^54^, and the genome annotation files have been submitted to Figshare dataset^55^.

## Technical Validation

### Completeness assessment of the assembled T2T genome

**Fig. 3:**
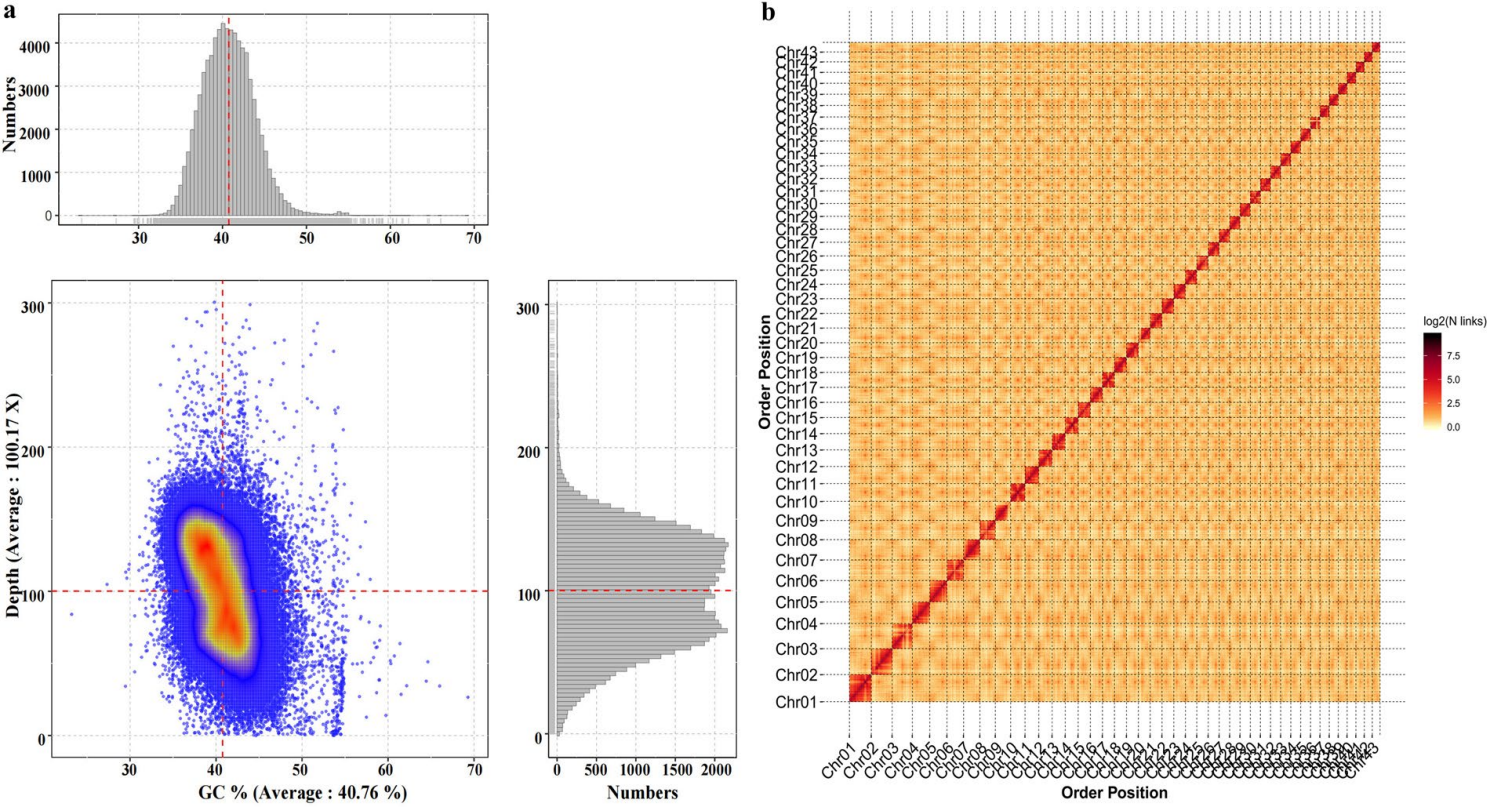
Completeness assessment of the *A. aspergillum* genome. (**a**) GC content and sequencing depth distribution density plot. The horizontal coordinate indicates the GC content and the vertical coordinate indicates the coverage depth. The right side shows the distribution of contig coverage depth, and the top side shows the distribution of GC content. The large figure in the center shows the scatter plot based on the GC distribution of contigs and the coverage depth information, in which the color shades are used to reflect the density of the points in the scatter plot. (**b**) The Hi-C interaction heatmap illustrates the quality of the Hi-C assembly and the interaction frequencies among 43 chromosomes in *A. aspergillum*. Chromosomes are represented by the squares. The strength of the interaction is defined by the color from yellow (low) to red (high).

To evaluate the completeness of the *A. aspergillum* genome assembly, we utilized Benchmarking Universal Single-Copy Orthologs (BUSCO)^56^ v5.2.2 (busco -m genome -c 24 -e 1e-3–Augustus) with the OrthoDB10 database to identify complete BUSCOs in the assembly. The BUSCO assessment identified a total of 954 BUSCO genes, of which 908 (95.18%) were completely captured, only six genes (0.63%) were fragmented, and 40 (4.19%) were missing from the genome, indicating the high integrity of the T2T genome assembly (Table 1 and S6). The percentage of complete BUSCOs was greater than that of *A. corticis* (91.2%) and *M. vulgaris* (94.3%) from Megascolecidae^16,17^, indicating that the integrity of the T2T assembly was higher than that of the chromosome-level assembly (Table 1). Additionally, bwa^57^ v0.7.10 (bwa index & & bwa mem -t 16) and Minimap2^58^ v2.24-r1122 (-I 20 G–MD -ax map-hifi/-I 20 G–MD -ax map-ont) software was used for aligning the Illumina short reads, CCS and ultra-long ONT reads to the assembled genome. The mapping rates of the Illumina, CCS, and ultra-long ONT reads were 99.72%, 99.86%, and 98.04%, respectively (Table S7). The average depth and coverage are shown in Tables S1, S7, and the sequencing data were analyzed for GC content and sample contamination (Fig. 3a). Moreover, the consensus quality value score of 46.89 obtained from the K-mer-based Merquery analysis^59^ (githup: https://github.com/marbl/merqury), indicated high accuracy of the T2T genome.

To examine the quality of Hi-C assembly and the interaction frequencies among different chromosomes, the genome was isotropically cut into 100 kb bins, and then the number of Hi-C Read Pairs between any two bins was used as a signal of the interaction between the two bins to make a Hi-C heatmap^60^. As shown in the Hi-C interaction heatmap, the strength of the correlation was higher at the diagonal position than at the non-diagonal position in each chromosome group (Fig. 3b).

**Fig. 4:**
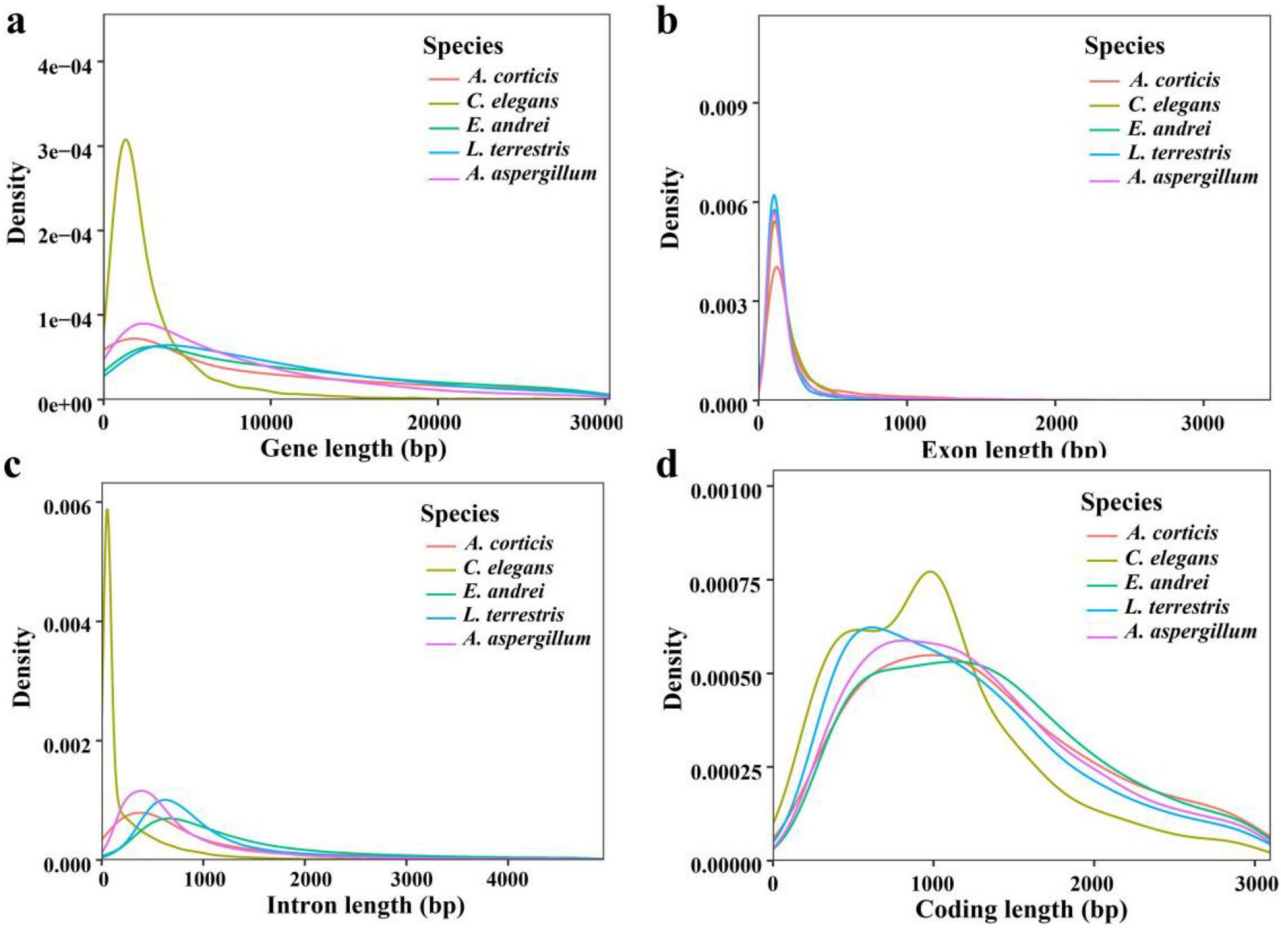
Comparison of the distribution of annotated gene features in *A. aspergillum* with four closely related species. (**a**) Gene length; (**b**) Exon length; (**c**) Intron length; (**d**) Coding length. The horizontal axis represents the length and the vertical axis represents the gene density.

To evaluate the gene prediction quality, accuracy, and reliability, we produced an annotated gene feature chart of the distribution of gene length, coding DNA sequence (CDS) length, exon length, and intron length in *A. aspergillum* with a model organism (*C. elegans*) and three closely related species (*A. corticis*, *E. andrei*, and *L. terrestris*). The consistent distribution among all closely related species further emphasized the ideal annotated gene dataset for *A. aspergillum* (Fig. 4). Complete orthologs for 95.60% of the conserved BUSCOs were identified, indicating high completeness of the predicted protein-coding genes.

## Supplementary information

Supporting material for Original article The high-quality telomere-to-telomere genome assembly of the earthworm (Amynthas aspergillum)

## Data Availability

All bioinformatics tools and pipelines were conducted by the prescribed guidelines from the respective manufacturer. The versions and corresponding parameters of software used in the study were described in the methods section. No custom package code was used during the analysis.
