# Supplementary material for: The high-quality telomere-to-telomere genome assembly of the earthworm (Amynthas aspergillum)
Source: Sci Data. 2025 Jun 2;12:931. doi: 10.1038/s41597-025-05058-w (PMC12130307; doi:10.1038/s41597-025-05058-w)
Supplement: Supplementary file 1 — Supporting material for Original article The high-quality telomere-to-telomere genome assembly of the earthworm (Amynthas aspergillum) [file 41597_2025_5058_MOESM1_ESM.pdf]

Supporting material for

Original article

The high-quality telomere-to-telomere genome assembly of the earthworm (*Amynthas aspergillum*)

Appendix

Table S1. Statistics of the sequencing data of the *A. aspergillum* genome.

Table S2. Assembly statistics of chromosomes.

Table S3. Functional annotated statistical results of *A. aspergillum*.

Table S4. Summary of RNA-sequencing..

Table S5. Statistics of other annotation result.

Table S6. The BUSCO assessment results of *A. aspergillum* genome.

Table S7. Alignment statistics of the T2T genome.

Fig. S1. The result of COI gene sequencing for the experimental sample.

Fig. S2. The result of karyotype analysis for *A. aspergillum* (2n=2x=86).

Fig. S3. The eggNOG function classification of the predicted sequence.

Table S1

Statistics of the sequencing data of the *A. aspergillum* genome.

Library type	Platform	Number of reads	Data Sizes (Gb)	Average Sequencing depth (×)	Average read length (bp)
Illumina	Illumina NOvaSeq 6000	475,151,866	71.00	92	150
CCS	PacBio Revio	5,172,062	80.34	101	15,534
ultralong ONT	PromethION	272,285	25.75	30	94,576
Hi-C	Illumina NOvaSeq 6000	873,323,762	130.81	171	150

Table S2

Assembly statistics of chromosomes.

Name	Length (bp)	Telomere number	Gap number
Chr01	30,867,748	2	0
Chr02	29,247,355	2	0
Chr03	28,391,443	2	1
Chr04	24,440,683	2	0
Chr05	24,147,203	2	0
Chr06	23,315,414	2	0
Chr07	22,919,233	2	0
Chr08	21,777,624	2	0
Chr09	21,347,434	2	0
Chr10	20,371,601	2	0
Chr11	19,365,930	2	0
Chr12	18,886,828	2	0
Chr13	18,417,096	1	1
Chr14	18,049,639	2	1
Chr15	17,124,882	2	0
Chr16	17,063,487	2	0
Chr17	17,000,192	2	0
Chr18	16,944,378	2	0
Chr19	16,721,379	2	0
Chr20	16,648,721	1	1
Chr21	16,586,161	2	0
Chr22	16,548,726	2	0
Chr23	16,269,298	2	0
Chr24	16,092,091	2	0
Chr25	15,956,968	2	0
Chr26	15,385,967	2	0
Chr27	14,792,995	2	0

Chr28	14.641.844	2	0
Chr29	14,467,141	2	0
Chr30	14,330,475	2	0
Chr31	14,269,266	2	0
Chr32	14,221,269	2	0
Chr33	14,132,398	2	0
Chr34	14,080,113	2	0
Chr35	14,071,238	2	0
Chr36	13,459,443	2	0
Chr37	12,935,041	2	0
Chr38	12,786,262	2	0
Chr39	12,488,920	1	0
Chr40	12,375,887	2	0
Chr41	11,879,937	2	0
Chr42	11,312,710	2	0
Chr43	10,820,067	2	0

Table S3

Functional annotated statistical results of *A. aspergillum*.

Database type	Number of genes	Percentage (%)
NR	29,244	81.86
eggNOG	23,332	65.31
GO	26,919	75.35
KEGG	25,075	70.19
TrEMBL	30,288	84.79
KOG	21,086	59.03
SWISS-PROT	20,045	56.11
Pfam	28,051	78.52
Annotated	31,657	88.62
Unannotated	4,066	11.38
Total	35,723	100

Table S4

Summary of RNA-sequencing.

Type	Number of reads	Data sizes (Gb)	Q20 (%)	Q30 (%)	GC (%)
RNA-seq	34,331,653	10.21	99.79	98.73	45.54

Table S5

Statistics of other annotation result.

Type	Text
tRNAs	899
rRNAs	2,734
miRNAs	0
snoRNAs	12
snRNAs	52
Pseudogene number	138
Pseudogene length (bp)	323,256
Motif	1,959
Domain	65,549

Table S6

The BUSCO assessment results of *A. aspergillum* genome.

BUSCO type	Number	Percentage (%)
Complete BUSCOs	908	95.18
Complete and single-copy BUSCOs	813	85.22
Complete and duplicated BUSCOs	95	9.96
Fragmented BUSCOs	6	0.63
Missing BUSCOs	40	4.19
Total lineage BUSCOs	954	100

Table S7

Alignment statistics of the T2T genome.

Data type	Number of mapped reads	Mapping rate (%)	Coverage (%)	Coverage (>=5X,%)	Coverage (>=10X,%)	Coverage (>=20X,%)
Illumina	473,806,001	99.72	99.81	99.46	99.00	97.66
CCS	5,164,628	99.86	99.94	99.72	99.46	98.50
ultralong ONT	266,944	98.04	99.61	99.40	98.73	83.42

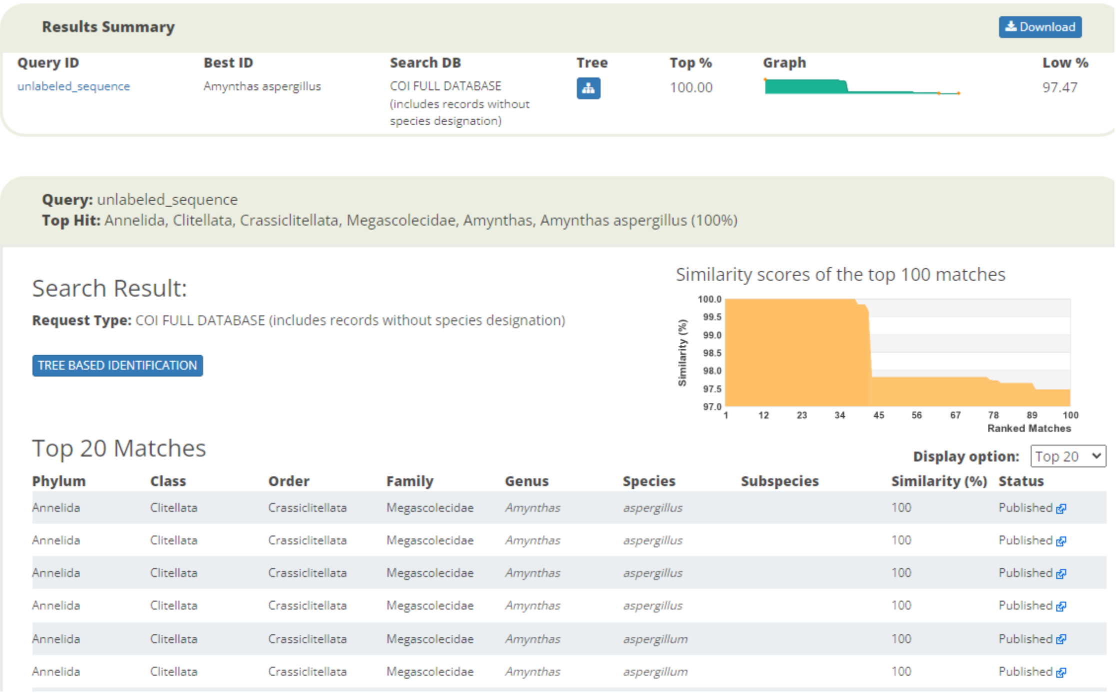

Fig. S1. The result of COI gene sequencing for the experimental sample. 100% species similarity with *Amynthas aspergillum*.

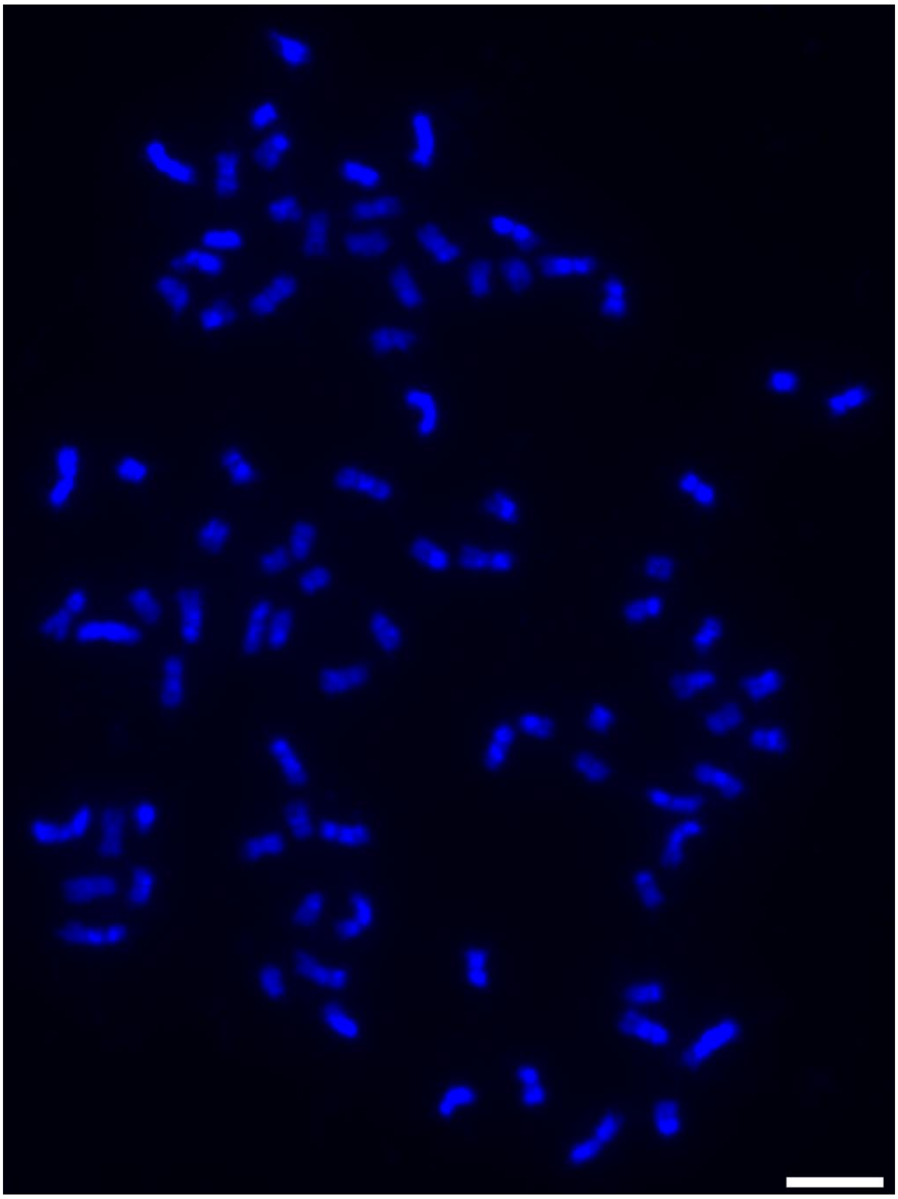

Fig. S2. The result of karyotype analysis for *A. aspergillum* (2n=2x=86). The blue patterns in the image represent chromosomes. Scale bar, 5 μm.

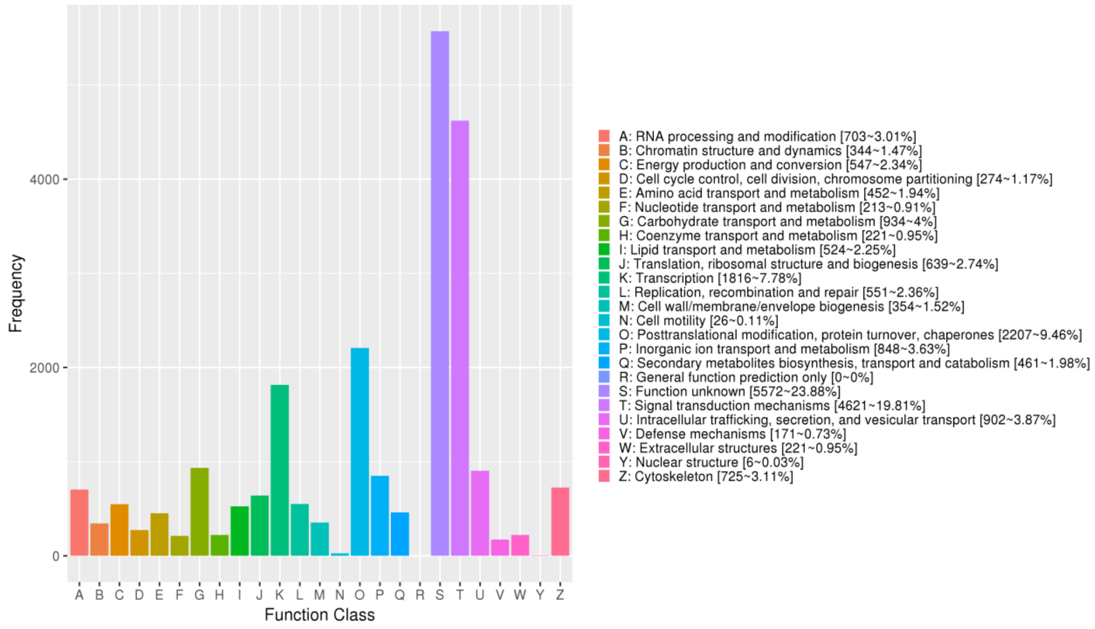

Fig. S3. The eggNOG function classification of the predicted sequence. The horizontal axis represents the content of each classification of eggNOG and the vertical axis represents the number of corresponding genes.
